# A green approach to the synthesis of novel “Desert rose stone”-like nanobiocatalytic system with excellent enzyme activity and stability

**DOI:** 10.1038/srep06606

**Published:** 2014-10-13

**Authors:** Min Wang, Wen-Jing Bao, Jiong Wang, Kang Wang, Jing-Juan Xu, Hong-Yuan Chen, Xing-Hua Xia

**Affiliations:** 1 State Key Laboratory of Analytical Chemistry for Life Science, School of Chemistry and Chemical Engineering, Nanjing University, Nanjing 210093, China

## Abstract

3D hierarchical layer double hydroxides (LDHs) have attracted extensive interest due to their unique electronic and catalytic properties. Unfortunately, the existing preparation methods require high temperature or toxic organic compounds, which limits the applications of the 3D hierarchical LDHs in biocatalysis and biomedicine. Herein, we present a green strategy to synthesize “Desert Rose Stone”-like Mg-Al-CO_3_ LDH nanoflowers *in situ* deposited on aluminum substrates *via* a coprecipitation method using atmospheric carbon dioxide. Using this method, we construct a novel “Desert Rose Stone”-like nanobiocatalytic system by using HRP as the model enzyme. Compared with the free HRP, the HRP/Mg-Al-LDH nanobiocatalytic system exhibits higher catalytic activity and stability. A smaller apparent Michaelis-Menten constant (0.16 mM) of this system suggests that the encapsulated HRP shows higher affinity towards H_2_O_2_.

Owing to their large surface area, high mechanical strength and good biocompatibility, nanomaterials (such as carbon materials, inorganic minerals, metal-organic frameworks, etc.) have emerged as a new class of promising candidates for applications inbiomedicine^1^ and nanobiocatalysis^2^,^3^,^4^,^5^. Nanobiocatalytic systems could be constructed by incorporating enzymes into nanostructured materials by various strategies. Compared with the free enzymes, the enzymes in nanobiocatalytic systems usually exhibit the enhanced enzymatic stability. In addition, the incorporated enzymes could be easily separated from their substrates and products and thus present the improved reusability. However, it is worth noting that the catalytic performance of the incorporated enzymes is sensitive to the properties of nanomaterials and the immobilized methods^6^,^7^. Therefore, exploring the appropriate nanomaterials and immobilized methods is becoming a key and challenge for the applications of nanobiocatalytic systems in industrial biocatalysis.

Layer double hydroxides (LDHs) are known as an important class of anionic lamellar clays and could be generally described by the formula 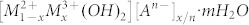 (where M^2+^ and M^3+^ are divalent and trivalent metal cations, and A^n−^ is an anion intercalated in the gallery). Due to their tunable composition, anion exchange ability, excellent adsorption capacity, good biocompatibility and stability, LDHs have been employed in a wide range of potential applications in various areas^8^,^9^,^10^,^11^,^12^. Until now, LDHs have been prepared by various methods such as coprecipitation^13^, ion exchange^14^, calcination-rehydration^15^, hydrothermal^16^ and electrochemistry^17^. The morphology and size of LDHs influencing their multifunctional properties and applications can be tailored by various environmental factors. However, LDHs prepared by conventional methods are usually stone-like or plate-like^18^,^19^ and their unwelcome surface areas urgently need to be improved for their potential applications. Recently, 3D hierarchical LDHs have attracted extensive interest due to their unique optical, electronic, magnetic, catalytic and absorption properties^20^,^21^,^22^,^23^,^24^,^25^,^26^,^27^. Various 3D hierarchical M-Al-LDHs with tunable architecture have been fabricated by calcination-rehydration or hydrothermal methods^20^,^21^,^22^. Especially, a hierarchical tri-metal Mg/Ni/Al-LDH nanoflowers on Al-foam was synthesized by an *in situ* multistep crystallization technique at 70°C^23^. Unfortunately, these methods usually require high reaction temperature, long aging time, complicated operating or organic compounds (such as ethylene glycol^22^, CTAB^28^), which greatly limits the application of 3D hierarchical LDHs in biocatalysis and biomedicine. Therefore, exploiting a novel and green strategy to fabricate the 3D hierarchical LDHs may shed lights on their potential applications in nanobiocatalytic systems with excellent performance.

Herein, we demonstrate an environmentally friendly approach to the synthesis of “Desert Rose Stone”-like Mg-Al-CO_3_ layer double hydroxide (Mg-Al-LDH) nanoflowers *via* a coprecipitation method as shown in Figure 1. Atmospheric CO_2_ in air is used as a source of CO_3_^2−^ and Al^3+^ in the form of Al(OH)_3_ originated from the dissolution of aluminum sheet under mild alkaline conditions. The *in situ*-grown 3D hierarchical Mg-Al-LDH nanoflowers exhibit larger specific surface area than traditional plate-like LDHs. The proposed coprecipitaion method does not rely on high temperature or organic compounds and thus can be used for constructing nanobiocatalytic systems by one-step loading of biomolecules. As demonstration, a nanobiocatalytic system (HRP/Mg-Al-LDH nanoflowers) using horseradish peroxidase (HRP) as the model enzyme is constructed, showing excellent biocatalytic activity.

## Results

### Synthesis and characterization of Mg-Al-LDH and HRP/Mg-Al-LDH nanoflowers

**Figure 2:**
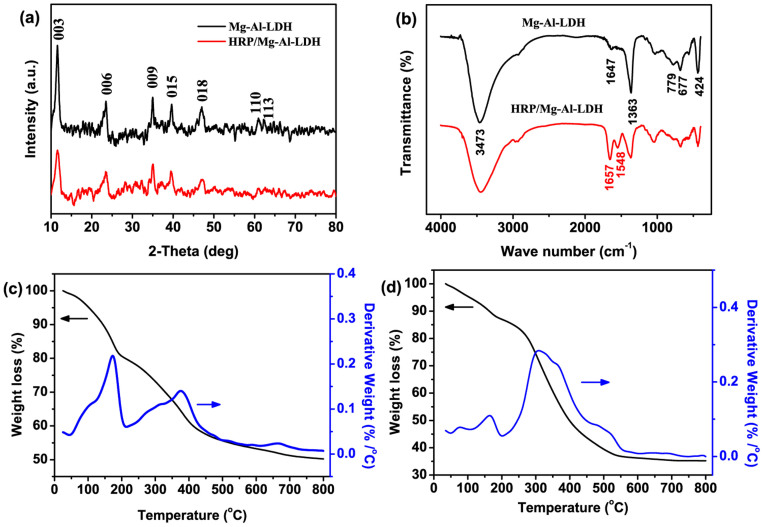
(a) XRD patterns and (b) FTIR spectra of the Mg-Al-LDH and HRP/Mg-Al-LDH nanoflowers. (c) and (d) TG-DTG curves of the Mg-Al-LDH and HRP/Mg-Al-LDH nanoflowers, respectively.

Mg-Al-LDH nanoflowers are synthesized in a solution of MgCl_2_ *via* the proposed coprecipitation approach. Atmospheric CO_2_ in air and aluminum sheet are used as the source of CO_3_^2−^ and Al^3+^ ions, respectively. HRP/Mg-Al-LDH nanoflowers are synthesized by the same procedure with the addition of HRP in solution. The XRD patterns of Mg-Al-LDH and HRP/Mg-Al-LDH nanoflowers are shown in Figure 2a. The occurrence of the characteristic reflections of a typical LDH with a series of (00*l*) peaks confirms the successful synthesis of LDHs. The peaks at 11.56°, 23.48° and 34.96° correspond to (003), (006) and (009) reflections, respectively. According to the Bragg formula nλ = 2dsinθ, the interlayer spacing of the as-prepared LDHs is calculated to be 0.765 nm, consistent with the value of Mg-Al-CO_3_ LDHs reported previously^22^. Compared with the Mg-Al-LDH nanoflowers, the intensity of diffraction peaks of HRP/Mg-Al-LDH nanoflowers decreases, which suggests a decreased crystallinity due to the introduction of HRP. In addition, no diffraction peaks are observed in the low-angle XRD pattern (0.5–10°) of HRP/Mg-Al-LDH nanoflowers (Figure S1), which demonstrates that the intercalation of HRP into LDH layers cannot occur within the immobilization process. The possible structure model of HRP/Mg-Al-LDH hybrids may be similar to the one reported by Vial *et al*^29^. The main interactions between HRP and LDHs are presumed to be hydrogen-bonding and/or electrostatic interactions since the HRP molecule (pI = 8.9^30^) carries positive charges at pH 10. The molar ratio of Mg and Al in LDHs obtained by ICP-AES is about 1.7:1 which is slightly lower than 2:1. The observed result may be ascribed to the contamination of aluminum compounds^31^.

The encapsulation of proteins into the layered nanostructures was also confirmed by infrared spectroscopic characterizations (Figure 2b). In the case of Mg-Al-LDH nanoflowers, the adsorption peaks around 3500 cm^−1^ is attributed to the O-H stretching modes of hydroxyl groups in LDH octahedron layers and interlayer water molecules. The weak peak at 1647 cm^−1^ is associated with the deformation mode of interlayer water molecules. The strong peak at 1363 cm^−1^ belongs to the vibration mode of CO_3_^2−^ in the gallery of LDHs. The peaks at 779, 677and 424 cm^−1^ are ascribed to the M-O stretching mode and the M-O-H deformation mode (M = Mg and Al). Compared with the Mg-Al-LDH, the FTIR spectrum for HRP/Mg-Al-LDH exhibits the characteristic amide I (1657 cm^−1^) and amide II (1548 cm^−1^) bands of proteins. These results confirm the successful preparation of Mg-Al-LDH and HRP/Mg-Al-LDH nanomaterials.

The thermal stability of the as-prepared LDH nanoflowers was also studied by thermal gravimetric analysis. The TG-DTG curves of Mg-Al-LDH and HRP/Mg-Al-LDH nanoflowers are shown in Figures 2c-d. Two distinct weight loss steps are observed for Mg-Al-LDH nanomaterials. The first step (25–195°C) with a mass loss of 18.95% corresponds to the removal of the absorbed water on the surface of LDHs and interlayer water. The second step (200–550°C) with a mass loss of 26.77% is ascribed to the dehydroxylation of the Mg-Al hydroxide layers and the decomposition of intercalated CO_3_^2−^ ions. Similar to Mg-Al-LDH, the HRP/Mg-Al-LDH nanoflowers also exhibit two typical weight loss steps with mass losses of 11.92% in the first step and 51.34% in the second step. It is worth noting that the mass loss in the second step for HRP-containing LDHs is significantly larger than that for the pure LDHs, which can be explained by that the dehydroxylation and anion decomposition of LDHs are accompanied by the decomposition of HRP molecules. The results further confirm that HRP has been successfully incorporated into the nano-biohybrid. According to the HRP concentration in the supernatant tested by UV-vis spectroscopy, the encapsulation efficiency of HRP (defined as the ratio of the amount in LDHs and the added total amount) is calculated as 85.2%.

**Figure 3:**
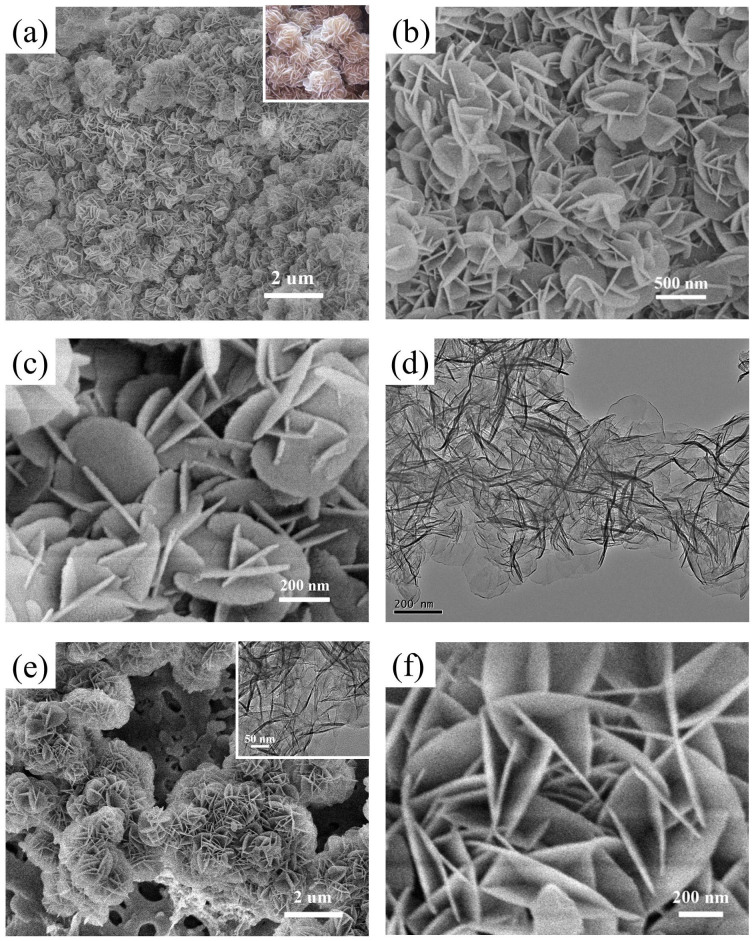
(a) Low-and (b–c) high-magnification SEM images, and (d) TEM image of the 3D hierarchical Mg-Al-LDH nanoflowers. (e) Low- and (f) high-magnification SEM images of the HRP/Mg-Al-LDH nanoflowers. Inset in Figure 2a shows the photo of the natural “Desert Rose Stone”. Inset in Figure 2e shows the TEM image of HRP/Mg-Al-LDH nanoflowers.

The typical SEM and TEM images of the as-prepared Mg-Al-LDH nanoflowers at different magnifications are shown in Figures 3a–d. Lots of Mg-Al-LDH particles with a diameter about 300 nm, whose structure is similar to “Desert Rose Stone”, could be observed from the low-magnification SEM image (Figure 3a). The high-magnification SEM images show that these LDH nanoparticles exhibit unique 3D hierarchical nanoflower structures constructed by plentiful intercalated round nanoplates with 300 nm in lateral size and 20 nm in thickness (Figures 3d–c). The TEM image indicates the presence of the nanoflowers with thin and smooth nanoplates (Figure 3d). The N_2_ adsorption-desorption isotherm of Mg-Al-LDH nanoflowers is shown in Figure S2. The BET surface area of the LDH nanoflowers is 79.4 cm^2^/g, which is larger than those of the conventional plate-like LDHs^32^,^33^. In addition, the as-prepared HRP/Mg-Al-LDH nanoflowers display the similar 3D nanoflower structures constructed by round nanoplates with 500 nm in diameter (Figures 3e–f). The TEM image of HRP/Mg-Al-LDH nanoflowers (Inset in Figure 3e) further confirms the presence of intercalated nanostructures.

**Figure 4:**
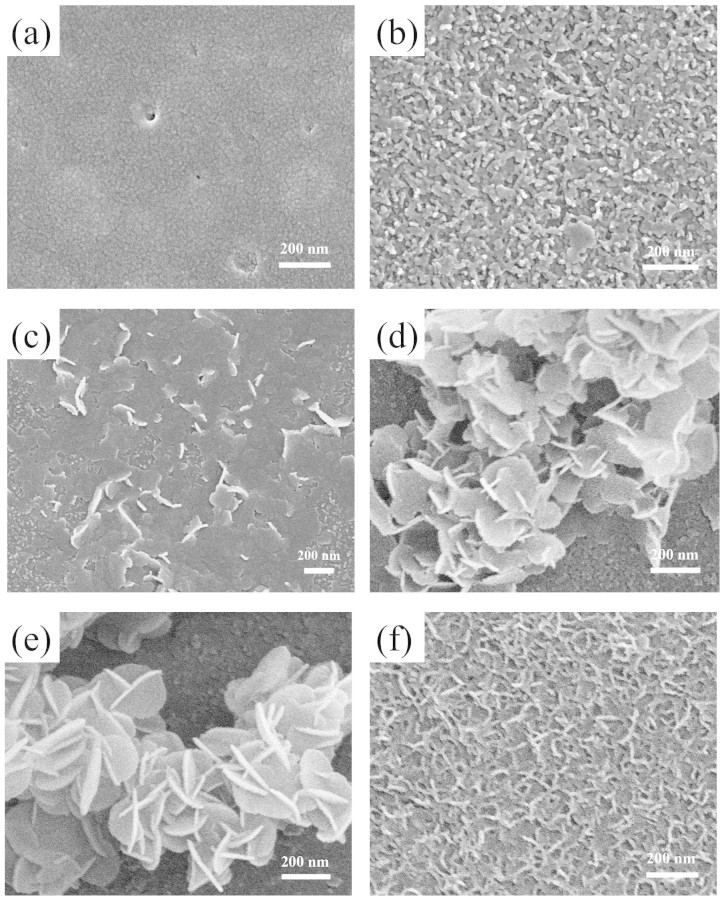
SEM images of the 3D hierarchical Mg-Al-LDH nanoflowers with different reaction times: (a) 0 h, (b) 30 min, (c) 1 h, (d) 2 h, (e) 5 h and (f) 20 h.

**Figure 5:**
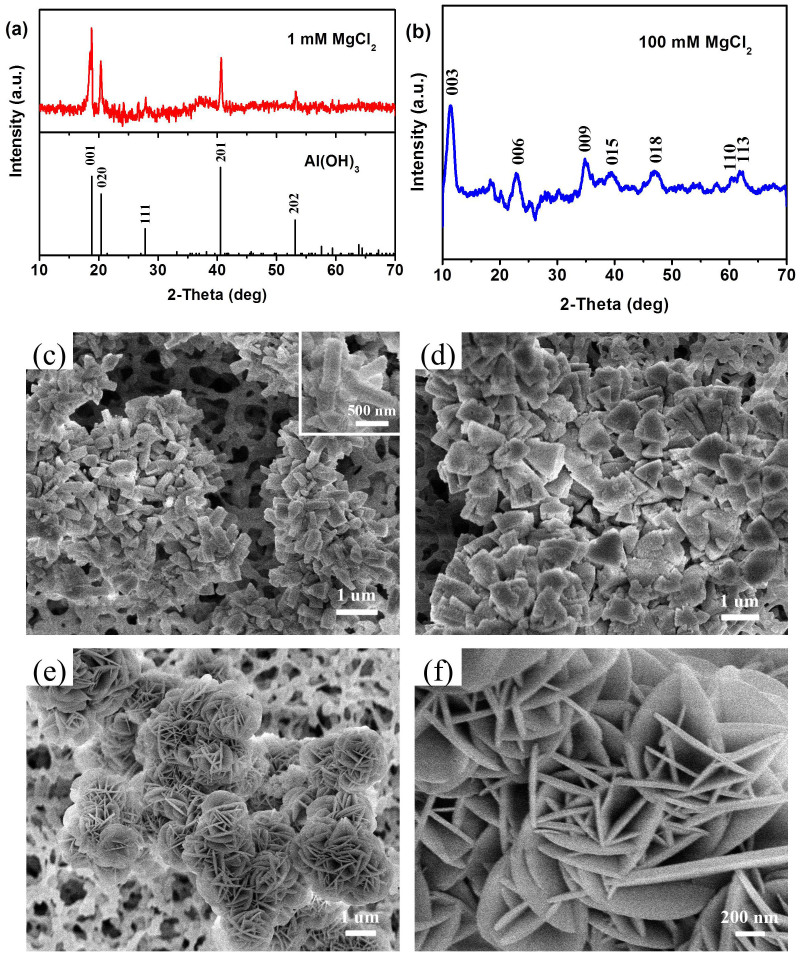
(a) XRD pattern and (c–d) SEM images of the precipitation formed from 1 mM MgCl_2_ precursor. Inset in Figure 3c shows the high-magnification SEM image of the precipitation. (b) XRD pattern and (e–f) SEM images of Mg-Al-LDHs formed from 100 mM MgCl_2_ precursor.

In order to investigate the formation process of 3D hierarchical nanoflowers, the morphology of aluminum substrate at different reaction intervals was characterized (Figure 4). The aluminum sheet exhibits a relatively smooth surface (Figure 4a) before the coprecipitation reaction. After reacted for 30 min, lots of nanoparticles can be observed at the surface of aluminum sheets (Figure 4b), which should be ascribed to the formation of the crystal nucleus of Mg-Al-CO_3_ LDHs. At this nucleation stage, Al(OH)_3_ is released continuously from the aluminum sheet and further reacted with Mg^2+^, OH^−^ and CO_3_^2−^ to form the LDH nucleus. The proposed reaction equations relating to the formation of LDH nanoflowers are shown in Equations (1–3). When the reaction time is prolonged, the small flakes appear (Figure 4c) and grow in the direction of the solution. Therefore, 3D hierarchical nanomaterials consisted of intercalated irregular nanoplates at the surface of aluminum sheet are formed (Figure 4d). At a reaction time of 5 h, the physical structure of the as-prepared nanomaterials tends to be the perfect one that the 3D hierarchical nanoflowers consist of plentiful regular round nanoplates (Figure 4e). After the gradual growth of nanoflowers with a certain height, the *in situ*-grown nanoflowers will fall off the aluminum sheet into solution, which makes the aluminum sheet to the nucleation stage (Figure 4f). Many literatures reported that 3D hierarchical LDHs could be prepared in the presence of organic compounds^22^,^28^. Fortunately, both organic compounds and high temperature are not required in the present approach. It is speculated that the possible reason of the formation of 3D nanoflowers is as follows: CO_3_^2−^ ions are derived from the atmospheric CO_2_ at pH 10 and react with Mg^2+^ and Al(OH)_3_ near the aluminum substrate surface. The distance-dependent concentration gradient of these ions at the surface of aluminum substrate may avoid the fast growth of LDHs^34^. Therefore, the Mg-Al-LDHs can grow in a certain orientation and self-assembled into the flower-like nanostructures. 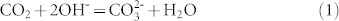

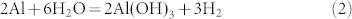

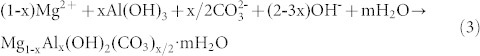
In addition, the effect of MgCl_2_ concentration on the formation of Mg-Al-LDH was further studied. Figures 5a–b show the XRD patterns of the precipitations obtained from 1 mM and 100 mM MgCl_2_ precursors, respectively. Compared with the standard card of Al(OH)_3_ (ICCD PDF no. 20-0011), the sample from 1 mM MgCl_2_ can be indexed to be the Al(OH)_3_ crystal (Figure 4a). This phenomenon is explained by that, at low concentration of MgCl_2_, the Mg^2+^ ions are limited and hardly coprecipitated with Al(OH)_3_ to generate Mg-Al-LDHs. As shown in Figures 5c–d, the Al(OH)_3_ precipitation displays the rod-shaped or triangular nanostructures with 600–800 nm in lateral size. At high MgCl_2_ concentration (100 mM), the obtained sample exhibits the characteristic reflections of a LDH phase, which indicates the successful synthesis of Mg-Al-CO_3_ LDHs (Figure 4b). As shown in Figures 5e–f, the as-prepared LDHs exhibit the similar 3D hierarchical nanostructures with larger nanoplates in lateral size and thickness compared with the LDHs formed from 20 mM MgCl_2_. These results demonstrate that a higher MgCl_2_ concentration will result in a larger concentration gradient of Mg^2+^ ions at the surface of aluminum substrate, which accelerates the growth of LDH nanoflowers. As a result, the larger LDH nanoflowers are obtained.

### Enzymatic activity of the HRP/Mg-Al-LDH nanobiocatalytic system

**Figure 6:**
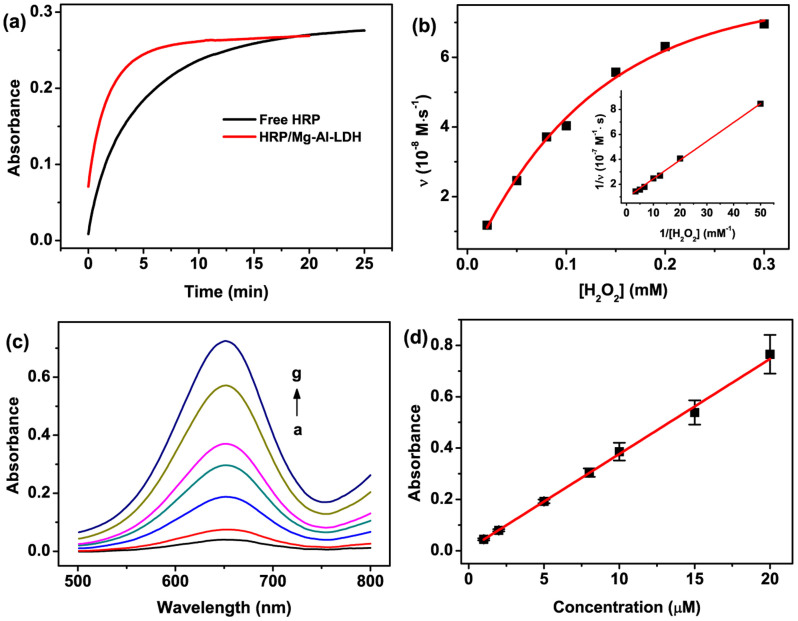
(a) Time-dependent absorbance changes at 652 nm in the presence of 0.1 mM TMB and 20 µM H_2_O_2_ catalyzed by free HRP and HRP/Mg-Al-LDH nanoflowers. (b) Plots of the initial reaction velocity versus the concentration of H_2_O_2_ for HRP/Mg-Al-LDH nanoflowers. The TMB concentration was 0.1 mM. Inset: Lineweaver-Burk plots of HRP/Mg-Al-LDH nanoflowers. (c) UV-vis spectra of TMB_ox_ in the presence of 0.1 mM TMB and different H_2_O_2_ concentration for HRP/Mg-Al-LDH nanoflowers, a-g: 1, 2, 5, 8, 10, 15 and 20 µM. (d) Plots of the absorbance at 652 nm versus H_2_O_2_ concentration.

Nanobiocatalytic systems based on nanostructured materials have attracted increasing attention due to their potential applications in biofuel cells, antifouling and proteomic analysis^35^,^36^. In the present work, HRP, as a model enzyme, has been incorporated into the LDH nanoflowers by the proposed coprecipitation method to construct a “desert rose stone”-like HRP/Mg-Al-LDH nanobiocatalytic system. H_2_O_2_ and TMB are chosen as the model substrates for HRP. In the presence of H_2_O_2_, HRP can catalyze the oxidation of TMB to generate TMB_ox_ which exhibits two absorption bands centered at 370 nm and 652 nm. Therefore, the catalytic activity of HRP can be easily evaluated by monitoring the absorbance at 652 nm in time-scan mode. Figure 6a shows the typical kinetic curves for free HRP and HRP/Mg-Al-LDH nanobiocatalytic systems. The final concentration of HRP is 1 ng/mL for each biocatalytic system. Compared with the free HRP system, the HRP/Mg-Al-LDH nanobiocatalytic system exhibits a much higher catalytic reaction velocity, which demonstrates that the catalytic activity of HRP incorporated into the Mg-Al-LDH nanoflowers is greatly enhanced. It is speculated that the enhanced enzyme activity may be ascribed to the conformation change of the immobilized HRP, which further results in an increase in the proportion of the exposed active sites.

In general, the amide I band in FTIR spectrum is sensitive to the changes of secondary structure in proteins and thus is usually used to monitor the conformation change of proteins. The contents of secondary structure can be calculated by integrating the distinct Gaussian peaks^37^,^38^,^39^. The FTIR spectrum of free HRP in 4000–400 cm^−1^ is shown in Figure S3. In order to prove the above speculation, the amide I bands for free HRP and the immobilized HRP are deconvoluted (Figure S4). In the amide I band, the bands at 1622, 1632 and 1695 cm^−1^ are assigned to β-sheet components; the bands at 1642, 1657 and 1678 cm^−1^ are attributed to random coil, α-helix and β-turn structures, respectively (Table S1)^40^. The contents of each secondary structure for free HRP and HRP immobilized in Mg-Al-LDH nanoflowers are shown in Table 1. After incorporated into Mg-Al-LDH nanoflowers, the content of α-helix structure of HRP molecules increases from 34.62% to 37.34%, which is accompanied by an decrease of the amount of β-structure (β-sheet and β-turn, from 55.12% to 47.38%)^41^. The change of secondary structure compositions reflects the change of protein conformation. These phenomena indicate that the conformation of HRP is changed due to the hydrogen-bonding and/or electrostatic interactions with LDH nanoflowers after HRP is incorporated into the nano-biohybrid.

Affinity of enzymes toward the substrates can be evaluated by Michaelis-Menten constant *K*_m_. The smaller Michaelis-Menten constant indicates the higher affinity. The Michaelis-Menten constant *K*_m_ and the maximum reaction velocity *υ*_max_ can be estimated from the Lineweaver-Burke equation^41^: 
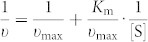
. The typical Michaelis-Menten plot (Figure 6b) and Lineweaver-Burke plot (Inset in Figure 6b) of the HRP/Mg-Al-LDH nanobiocatalytic system are obtained by monitoring the absorbance change at 652 nm in the presence of different H_2_O_2_ concentration (Figure S5). Based on the slope and intercept of the Lineweaver-Burke plot, the apparent *K*_m_ and *υ*_max_ are calculated to be 0.16 mM and 1.02 × 10^−7^ M/s, respectively. It is obvious that the apparent *K*_m_ of the proposed nanobiocatalytic system is smaller than that of free HRP (3.7 mM) and other reported systems such as HRP/Titania Sol-Gel Matrix (1.89 mM)^42^, HRP/CMCS-Au NPs (0.57 mM)^43^, HRP/ZnO-GNPs-Nafion (1.76 mM)^44^. These results demonstrate that the “Desert Rose Stone”-like HRP/Mg-Al-LDH nanobiocatalytic system shows higher affinity toward H_2_O_2_.

### Detection of H_2_O_2_ by the HRP/Mg-Al-LDH nanobiocatalytic system

In the present work, a simple and sensitive H_2_O_2_ biosensor was constructed based on the high affinity of HRP/Mg-Al-LDH nanoflowers to H_2_O_2_. Figure 6c shows the UV-vis spectra of TMB_ox_ in the presence of different H_2_O_2_ concentration. As the H_2_O_2_ concentration increases, the absorbance at 652 nm increases accordingly (Figure 6d). The proposed biosensor shows a linear response to H_2_O_2_ in the concentration range from 1 to 20 µM with a detection limit of 0.34 µM, which is better than those of the HRP-based biosensors^42^,^45^. In addition, the stability of the proposed biosensor was also studied. After the storage for 72 h at room temperature, the HRP/Mg-Al-LDH nanobiocatalytic system retains 80.3% of the initial activity, while the free HRP system retains only 4.4% of its initial activity. The results suggest that the HRP/Mg-Al-LDH nanobiocatalytic system exhibits higher stability than the free HRP system.

## Discussion

We propose a green coprecipitation method for the facile synthesis of “desert rose stone”-like 3D hierarchical Mg-Al-CO_3_ LDH nanoflowers. The growth process of the as-prepared3D hierarchical LDH nanoflowers has been investigated. The slow diffusion of CO_2_ from air is considered to be a key process for tuning the morphology of LDH nanoflowers, which avoids the fast growth of LDHs. The proposed coprecipitaion method does not require high temperature or organic compounds, and can be used for preparing bio-nanomaterials. The prepared “Desert Rose Stone”-like HRP/Mg-Al-LDH nanoflowers exhibit higher catalytic activity and stability than the free HRP, which suggests the LDH nanoflowers show good biocompatibility. The encapsulation of biomolecules is not limited to HRP. Biomolecules including individual and multiple enzymes, proteins and microRNA can also be incorporated to prepare nanobiofunctional systems for biocatalysis and biomedicine.

## Materials

Horseradish peroxidase (HRP, 261 U/mg) and 3,3′,5,5′-Tetramethylbenzidine dihydrochloride hydrate (TMB) were purchased from Sigma-Aldrich (USA). Al sheet (purity 99.99%) was purchased from Xinjiang Zhonghe Limited Corp (Xinjiang, China). Phosphate buffer solution (PBS, 25 mM, pH 7.0) was prepared by mixing stock solutions of K_2_HPO_4_ and KH_2_PO_4_. All other reagents were of analytical grade and used as received. All the solutions were prepared by using ultrapure water (18.3 MΩ·cm) from the Millipore Milli-Q system.

## Methods

### Synthesis of the Mg-Al-LDH and HRP/Mg-Al-LDH nanoflowers

The Mg-Al-LDH nanoflowers were synthesized in moderate conditions. Briefly, the pH of MgCl_2_ (20 mM) aqueous solution was adjusted to *ca.* 10 using aqueous NaOH (0.5 M) solution. Then, a small piece of Al sheet was put into the solution. The reaction vessel was covered with perforated parafilm to allow CO_2_ from air to diffuse into the system. After 18 h at room temperature, the obtained white precipitation (Mg-Al-LDH nanoflowers) was centrifuged and thoroughly washed with ultrapure water. The HRP/Mg-Al-LDH nanoflowers were synthesized by the same procedure with the addition of HRP (the final concentration 1.25 mg/mL) in MgCl_2_ aqueous solution. The fresh suspension was used for the analysis of enzyme activity and the dried power was used for characterizations. The HRP concentration in the supernatant was determined by the absorbance at 403 nm (ε_403_ = 9.1 × 10^4^ L·mol^−1^·cm^−1^)^46^, which allows us to calculate the encapsulation yield of HRP in nanoflowers. The encapsulation efficiency of HRP in Mg-Al-LDH nanoflowers is defined as the ratio of encapsulation yield to the initial weight of HRP.

### Kinetic Analysis and bioassay

Kinetic measurements were carried out at room temperature with 5 µL HRP/Mg-Al-LDH suspension or HRP solution in 500 µL PBS (25 mM, pH 5.0) containing 0.1 mM TMB and 20 µM H_2_O_2_. The absorbance at 652 nm in time-scan mode was monitored. The final concentration of HRP was 1 ng/mL for each biocatalytic system. Detection of H_2_O_2_ was finished as follows. Briefly, 5 µL HRP/Mg-Al-LDH suspension was added into 500 µL PBS (25 mM, pH 5.0) containing 0.1 mM TMB and different concentration of H_2_O_2_, followed by the reaction at 30°C for 20 min. The absorbance of the resulted solution at 652 nm was measured.

### Characterization

UV-vis absorption spectra were obtained on a UV 3600 spectrophotometer (Shimazu, Japan).Fourier transform infrared (FTIR) spectra were collected on a Nicolet 6700 FTIR spectrometer (Nicolet, USA).X-ray diffraction (XRD) measurement was performed on a XRD-6000 diffractometer (Shimadzu, Japan), using Cu Kα radiation (λ = 1.5418 Å) with a scan rate 2 deg/min. Scanning electron microscopy (SEM) images were obtained on a Hitachi S-4800 field-emission scanning electron microscope (Hitachi, Japan).Transmission electron microscopy (TEM) images were collected with a JEM-2100 high-resolution transmission electron microscope (JEOL, Japan). The molar ratio of Mg^2+^/Al^3+^ in Mg-Al-LDH was determined by an optima 5300DV inductively coupled plasma atomic emission spectroscopy (ICP-AES, Perkin Elmer, USA). Thermogravimetry- differential scanning calorimetry (TG-DSC) analysis was performed on a STA 449C thermal analyzer (Netzsch, Germany) under the air flow with a heating rate of 10 K/min. The N_2_ adsorption-desorption experiment was carried out by Micromeritics ASAP 2010 (Micromeritics, USA) at 77 K. Specific surface area of Mg-Al-LDH nanoflowers was calculated according to the Brunnauer-Emmett-Teller (BET) method.

## Author Contributions

M.W. performed experimental work on synthesis and studies of Mg-Al-LDHs and HRP/Mg-Al-LDHs. W.-J.B. and J.W. performed the morphology characterizations (TEM, SEM) and infrared spectroscopic characterizations. K.W., J.-J.X. and H.-Y.C. helped to analysis the experimental data. X.-H.X. led this project.

## Supplementary Material

Supplementary Information Supporting Information

## Figures and Tables

**Figure 1:**
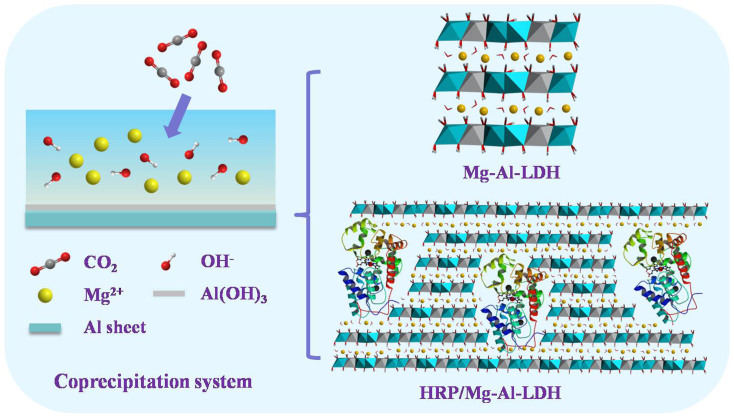
Illustration of the proposed green coprecipitation method for the synthesis of Mg-Al-LDH and HRP/Mg-Al-LDH nanoflowers.

**Table 1:** Secondary structure compositions of free HRP and HRP/Mg-Al-LDH nanoflowers

	Secondary structure compositions (%)			
	α-helix	β-sheet	β-turn	random coil
free HRP	34.62	23.52	31.60	10.26
HRP/Mg-Al-LDH	37.34	18.45	28.83	15.38

## References

[b1] Xia Y. Nanomaterials at work in biomedical research. Nat. Mater. 7, 758–760 (2008). 18813296 10.1038/nmat2277

[b2] Lykourinou V. *et al.* Immobilization of MP-11 into a mesoporous metal-organic framework, MP-11@ mesoMOF: a new platform for enzymatic catalysis. J. Am. Chem. Soc. 133, 10382–10385 (2011). 21682253 10.1021/ja2038003

[b3] Hua B. Y. *et al.* Greatly improved catalytic activity and direct electron transfer rate of cytochrome c due to the confinement effect in a layered self-assembly structure. Chem. Commun. 48, 2316–2318 (2012). 10.1039/c2cc17516a 22261736

[b4] Zhou Z. & Hartmann M. Progress in enzyme immobilization in ordered mesoporous materials and related applications. Chem. Soc. Rev. 42, 3894–3912 (2013). 23570038 10.1039/c3cs60059a

[b5] Su S. *et al.* Direct electrochemistry of glucose oxidase and a biosensor for glucose based on a glass carbon electrode modified with MoS_2_ nanosheets decorated with gold nanoparticles. Microchim. Acta 1–7 (2014).

[b6] Puddu V. & Perry C. C. Interactions at the silica-peptide interface: the influence of particle size and surface functionality. Langmuir 30, 227–233 (2013). 24328428 10.1021/la403242f

[b7] Pessela B. C. *et al.* Modulation of the catalytic properties of multimeric β-galactosidase from *E. coli* by using different immobilization protocols. Enzyme Microb. Technol. 40, 310–315 (2007).

[b8] Kapoor M. P. & Matsumura Y. Liquid-phase methanol carbonylation catalyzed over tin promoted nickel-aluminium layered double hydroxide. Catal. Today 93, 287–291 (2004).

[b9] Davis R. J. & Derouane E. G. A non-porous supported-platinum catalyst for aromatization of n-hexane. Nature 349, 313–315 (1991).

[b10] Lv L., He J., Wei M., Evans D. & Zhou Z. Treatment of high fluoride concentration water by MgAl-CO_3_ ayered double hydroxides: Kinetic and equilibrium studies. Water Res. 41, 1534–1542 (2007). 17316743 10.1016/j.watres.2006.12.033

[b11] Camino G., Maffezzoli A., Braglia M., De Lazzaro M. & Zammarano M. Effect of hydroxides and hydroxycarbonate structure on fire retardant effectiveness and mechanical properties in ethylene-vinyl acetate copolymer. Polym. Degrad. Stabil. 74, 457–464 (2001).

[b12] Li B., He J., G Evans D. & Duan X. Inorganic layered double hydroxides as a drug delivery system-intercalation and in vitro release of fenbufen. Appl. Clay Sci. 27, 199–207 (2004).

[b13] Bonnet S. *et al.* Synthesis of hybrid organo-mineral materials: anionic tetraphenylporphyrins in layered double hydroxides. Chem. Mater. 8, 1962–1968 (1996).

[b14] Meyn M., Beneke K. & Lagaly G. Anion-exchange reactions of layered double hydroxides. Inorg. Chem. 29, 5201–5207 (1990).

[b15] Géraud E., Prévot V., Ghanbaja J. & Leroux F. Macroscopically ordered hydrotalcite-type materials using self-assembled colloidal crystal template. Chem. Mater. 18, 238–240 (2006).

[b16] Clause O., Gazzano M., Vaccari A. & Zatorski L. Preparation and thermal reactivity of nickel/chromium and nickel/aluminium hydrotalcite-type precursors. Appl. Catal. 73, 217–236 (1991).

[b17] Yarger M. S., Steinmiller E. M. & Choi K. S. Electrochemical Synthesis of Zn-Al Layered Double Hydroxide (LDH) Films. Inorg. Chem. 47, 5859–5865 (2008). 18533629 10.1021/ic800193j

[b18] Liu Z. *et al.* Synthesis, anion exchange, and delamination of Co-Al layered double hydroxide: assembly of the exfoliated nanosheet/polyanion composite films and magneto-optical studies. J. Am. Chem. Soc. 128, 4872–4880 (2006). 16594724 10.1021/ja0584471

[b19] Klemkaite K., Prosycevas I., Taraskevicius R., Khinsky A. & Kareiva A. Synthesis and characterization of layered double hydroxides with different cations (Mg, Co, Ni, Al), decomposition and reformation of mixed metal oxides to layered structures. Cent. Eur. J. Chem. 9, 275–282 (2011).

[b20] Tao Q., Zhang Y., Zhang X., Yuan P. & He H. Synthesis and characterization of layered double hydroxides with a high aspect ratio. J. Solid State Chem. 179, 708–715 (2006).

[b21] Shao M. *et al.* Core-shell layered double hydroxide microspheres with tunable interior architecture for supercapacitors. Chem. Mater. 24, 1192–1197 (2012).

[b22] Yu X. Y. *et al.* Three-dimensional hierarchical flower-like Mg-Al-layered double hydroxides: highly efficient adsorbents for As (V) and Cr (VI) removal. Nanoscale 4, 3466–3474 (2012). 22538297 10.1039/c2nr30457k

[b23] Sun Z. *et al.* Fabrication and characterization of hierarchical Mg/Ni/Al layered double hydroxide framework on aluminum foam. Mater. Lett. 113, 83–86 (2013).

[b24] Prevot V., Caperaa N., Taviot-Gueho C. & Forano C. Glycine-assisted hydrothermal synthesis of NiAl-layered double hydroxide nanostructures. Cryst. Growth Des. 9, 3646–3654 (2009).

[b25] Wang H., Fan G., Zheng C., Xiang X. & Li F. Facile Sodium Alginate Assisted Assembly of Ni-Al Layered Double Hydroxide Nanostructures. Ind. Eng. Chem. Res. 49, 2759–2767 (2010).

[b26] Yang Y., Fan G. & Li F. Synthesis of novel marigold-like carbonate-type Mg-Al layered double hydroxide micro-nanostructures via a two-step intercalation route. Mater. Lett. 116, 203–205 (2014).

[b27] Yu J., Fan G., Yang Y. & Li F. Multi-level three-dimensional Mg-Al layered double hydroxide hierarchical microstructures with enhanced basic catalytic property. J, Colloid Interf. Sci. 432, 1–9 (2014). 10.1016/j.jcis.2014.06.054 25036381

[b28] Sun L. & Hu C. Facile synthesis *via* a solvothermal route and characterization of Mg-Al layered double hydroxide (LDH) 3D micro-nano structures. Mater. Res. Bull. 46, 1922–1927 (2011).

[b29] Vial S. *et al.* Nanohybrid-layered double hydroxides/urease materials: Synthesis and application to urea biosensors. Mater. Sci. Eng. C 26, 387–393 (2006).

[b30] Zhang Q. *et al.* Fabrication of a biocompatible and conductive platform based on a single-stranded DNA/graphene nanocomposite for direct electrochemistry and electrocatalysis. Chem. Eur. J. 16, 8133–8139 (2010). 20583058 10.1002/chem.201000684

[b31] Khan A. I. & O'Hare D. Intercalation chemistry of layered double hydroxides: recent developments and applications. J. Mater. Chem. 12, 3191–3198 (2002).

[b32] Shao M. F. *et al.* Core-shell layered double hydroxide microspheres with tunable interior architecture for supercapacitors. Chem. Mater. 24, 1192–1197 (2012).

[b33] Yang M. *et al.* Mg/Al-CO_3_ layered double hydroxide nanorings. J. Mater. Chem. 21, 14741–14746 (2011).

[b34] Noorduin W. L., Grinthal A., Mahadevan L. & Aizenberg J. Rationally designed complex, hierarchical microarchitectures. Science 340, 832–837 (2013). 23687041 10.1126/science.1234621

[b35] Kim J., Grate J. W. & Wang P. Nanobiocatalysis and its potential applications. Trends Biotechnol. 26, 639–646 (2008). 18804884 10.1016/j.tibtech.2008.07.009

[b36] Ansari S. A. & Husain Q. Potential applications of enzymes immobilized on/in nano materials: A review. Biotechnol. Adv. 30, 512–523 (2012). 21963605 10.1016/j.biotechadv.2011.09.005

[b37] Dong A., Huang P. & Caughey W. S. Protein secondary structures in water from second-derivative amide I infrared spectra. Biochemistry 29, 3303–3308 (1990). 2159334 10.1021/bi00465a022

[b38] Zhao H. Z., Sun J. J., Song J. & Yang Q. Z. Direct electron transfer and conformational change of glucose oxidase on carbon nanotube-based electrodes. Carbon 48, 1508–1514 (2010).

[b39] Bao W. J. *et al.* Distance-determined sensitivity in attenuated total reflection-surface enhanced infrared absorption spectroscopy: aptamer-Antigen compared to antibody-antigen. *Chem*. Commun. 50, 7787–7789 (2014). 10.1039/c4cc01920b 24901743

[b40] Al-Azzam W. *et al.* Structure of poly (ethylene glycol)-modified horseradish peroxidase in organic solvents: infrared amide I spectral changes upon protein dehydration are largely caused by protein structural changes and not by water removal per se. Biophys. J. 83, 3637–3651 (2002). 12496131 10.1016/S0006-3495(02)75364-2 PMC1302439

[b41] He H., Xu X., Wu H., Zhai Y. & Jin Y. In situ nanoplasmonic probing of enzymatic activity of monolayer-confined glucose oxidase on colloidal nanoparticles. Anal. Chem. 85, 4546–4553 (2013). 23531235 10.1021/ac4001805

[b42] Yu J. & Ju H. Preparation of porous titania sol-gel matrix for immobilization of horseradish peroxidase by a vapor deposition method. Anal. Chem. 74, 3579–3583 (2002). 12139071 10.1021/ac011290k

[b43] Xu Q., Mao C., Liu N.-N., Zhu J.-J. & Sheng J. Direct electrochemistry of horseradish peroxidase based on biocompatible carboxymethyl chitosan–gold nanoparticle nanocomposite. Biosens. Bioelectron 22, 768–773 (2006). 16600589 10.1016/j.bios.2006.02.010

[b44] Xiang C., Zou Y., Sun L.-X. & Xu F. Direct electrochemistry and enhanced electrocatalysis of horseradish peroxidase based on flowerlike ZnO–gold nanoparticle–Nafion nanocomposite. Sensor Acuat. B Chem. 136, 158–162 (2009).

[b45] Xiang C., Zou Y., Sun L.-X. & Xu F. Direct electrochemistry and enhanced electrocatalysis of horseradish peroxidase based on flowerlike ZnO-gold nanoparticle-Nafion nanocomposite. Sens. Actuat. B-Chem. 136, 158–162 (2009).

[b46] Roman R. & Dunford H. pH dependence of the oxidation of iodide by compound I of horseradish peroxidase. Biochemistry 11, 2076–2082 (1972). 5027617 10.1021/bi00761a013

