# Supplementary material for: A green approach to the synthesis of novel “Desert rose stone”-like nanobiocatalytic system with excellent enzyme activity and stability
Source: Sci Rep. 2014 Oct 13;4:6606. doi: 10.1038/srep06606 (PMC4194442; doi:10.1038/srep06606)
Supplement: Supplementary Information — Supporting Information [file srep06606-s1.pdf]

**Supporting information for**

# A green approach to the synthesis of novel "Desert rose stone"-like nanobiocatalytic system with excellent enzyme activity and stability

Min Wang, Wen-Jing Bao, Jiong Wang, Kang Wang, Jing-Juan Xu, Hong-Yuan Chen, Xing-Hua Xia*

State Key Laboratory of Analytical Chemistry for Life Science, School of Chemistryand Chemical Engineering, Nanjing University, Nanjing 210093, China.

*Corresponding author: xhxia@nju.edu.cn; Fax: +86-25-83685947

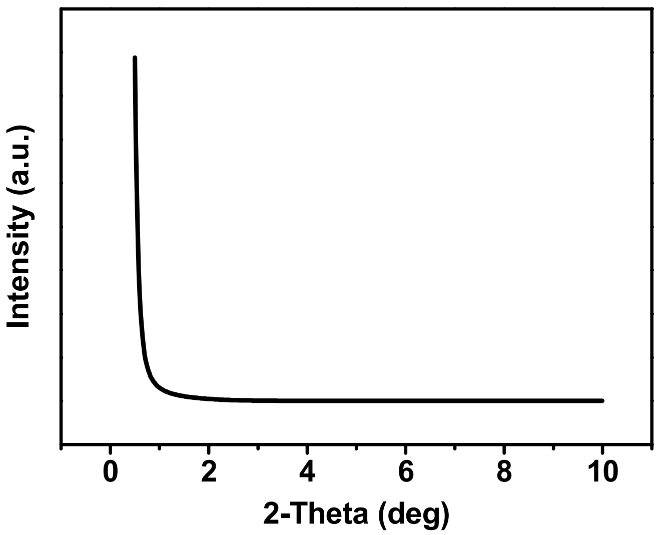


**Supplementary Figure 1** The low-angle XRD pattern of the HRP/Mg-Al-LDH nanoflowers.

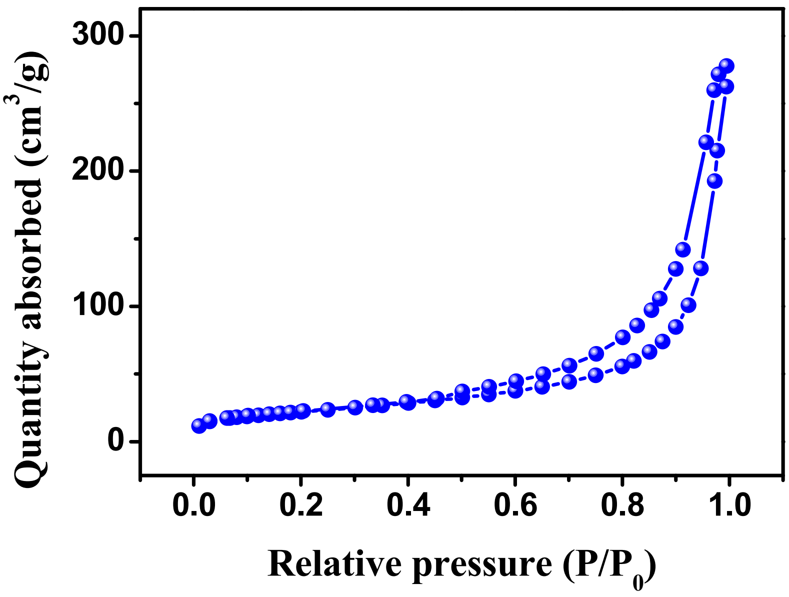


**Supplementary Figure 2** The $N_2$ adsorption-desorption isotherm of Mg-Al-LDH nanoflowers.

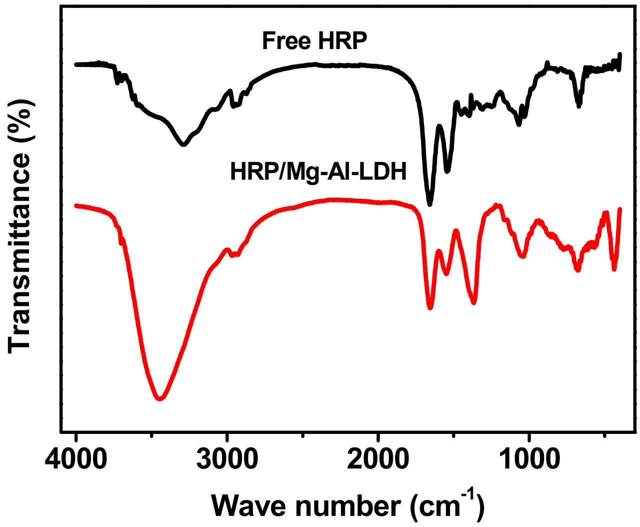


**Supplementary Figure 3** FTIR spectra of the free HRP and HRP/Mg-Al-LDH nanoflowers.

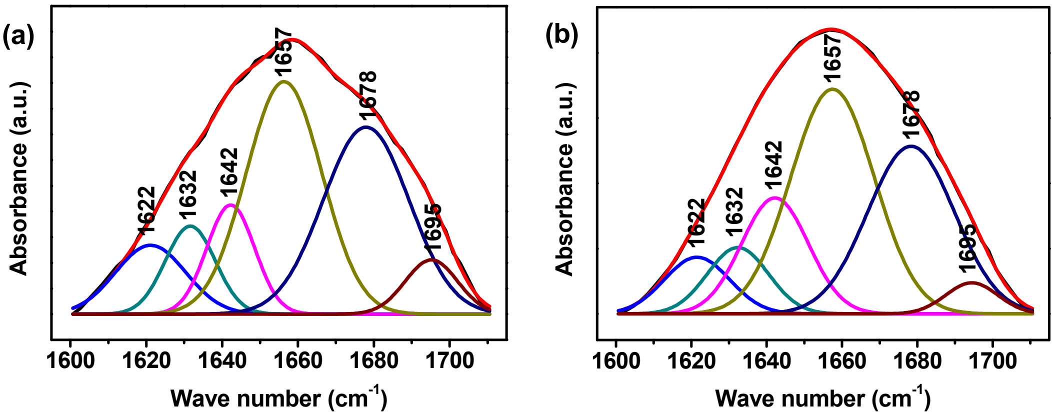


**Supplementary Figure 4** FTIR (black curve) and deconvoluted (other curves) spectra of free HRP (a) and HRP/Mg-Al-LDH (b) in the amide I region.

| Wave numbers ($cm^{-1}$) | Assignments |
|---|---|
| 1622, 1632, 1695 | β-sheet |
| 1642 | Random coil |
| 1657 | α-helix |
| 1678 | β-turn |

**Supplementary Table 1** Assignments of the deconvoluted peaks in Amide I band for HRP.

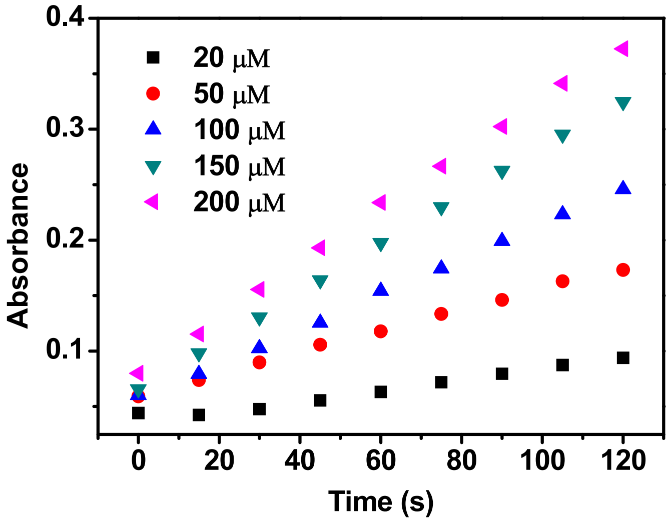


**Supplementary Figure 5** Time-dependent absorbance changes at 652 nm in the presence of 0.1 mM TMB and different $H_2O_2$ concentration catalyzed by HRP/Mg-Al-LDH nanoflowers.
